# Extraventricular Drain Care Bundle Decreases Cerebrospinal Fluid Infection Rates Associated With Extraventricular Drain-Related Procedures and Systemic Infection

**DOI:** 10.7759/cureus.52440

**Published:** 2024-01-17

**Authors:** Marissa Lozano, Alice S Wang, Imran Siddiqi, Opal Dy, Katherine Ko, Raed Sweiss, Dan E Miulli

**Affiliations:** 1 Neuro Intensive Care Unit, Arrowhead Regional Medical Center, Colton, USA; 2 Neurosurgery, Riverside University Health System Medical Center, Moreno Valley, USA; 3 Neurosurgery, Arrowhead Regional Medical Center, Colton, USA

**Keywords:** infection rates, systemic infection, bundle care, csf infection, evd

## Abstract

Background: Infection associated with extraventricular drain (EVD)-related procedures is well known.

Objective: To investigate the impact of our institution’s EVD care bundle on the infection rates associated with EVD-related procedures.

Methods: A retrospective study was conducted from June 2022 to June 2023 to compare the infection rate six months before and six months after the implantation of the EVD care bundle.

Results: A total of 58 patients were included in the study (n=33 patients in 2022 and n=25 patients in 2023). The infection rate was 21.2% (7/33) prior to the implementation of the EVD care bundle and 0.0% (0/25) afterward. The seven patients with cerebrospinal fluid (CSF) infection did not have a higher total number of EVD-related procedures compared to the other 26 patients without CSF infection (8.0 vs. 9.4, p=0.7364); however, the mean number of EVD replacements was higher in patients with CSF infection (1.4 vs. 3.4, p=0.0028). The total number of EVD-related procedures was not different between 2022 and 2023 (8.3 vs. 5.2, respectively, p=0.1892); however, the mean number of EVD replacements was lower in 2023 (1.8 vs. 1.0, p=0.0257). In 2022, 22/33 patients had systemic infection, among which 7/22 also had CSF infection. In 2023, 13/25 patients had a systemic infection, among which 0/13 had CSF infection.

Conclusions: The EVD care bundle consisting of standardizations, checklists, and monitoring reduces the CSF infection rates associated with EVD-related procedures and systemic infection.

## Introduction

Extraventricular drain (EVD) placement is a commonly performed bedside neurosurgical procedure. Indications may include hydrocephalus; suspected elevated intracranial pressure secondary to intracranial hematoma, neoplasm, or infection; or traumatic brain injury in selected trauma patients [1,2]. EVD allows for measurement of intracranial pressure and treatment of elevated intracranial pressure. This procedure is not without complications. Initial complications may include but are not limited to infection, hemorrhage, seizure, misplacement, dislodgement, and blockage which may lead to further complications such as ventriculitis, meningitis, brain abscess, or subdural empyema [3]. These “secondary” complications were found to be associated with longer hospitalization, morbidity, and mortality [4]. The complications may occur at the time of EVD insertion or after dressing changes, cerebrospinal fluid (CSF) sampling, or instillation of intrathecal medication.

At our institution, infections associated with EVD-related procedures were observed. Variabilities in techniques in EVD insertion, EVD replacement, CSF sampling, intrathecal medication installation, and dressing change were identified and considered to be related to an increased risk of infection. EVD care bundle studies have been shown to decrease the infection rate [5,6]. There was no EVD care bundle at our institution and we hypothesized that it could help decrease the infection rate. Therefore, to address this complication and to standardize care, collaboratively, team members from the neurosurgery, intensive care units, and nursing discussed and developed an EVD care bundle. The EVD care bundle consists of four standardization procedures, associated checklists, and monitoring requirements. The EVD care bundle is used for 1) EVD insertion, 2) EVD dressing change, 3) CSF sampling, and 4) intrathecal medication administration. The implementation of the EVD care bundle went into effect in January 2023. The purpose of this study was to investigate whether our institution’s EVD care bundle affected the infection rate associated with EVD.

## Materials and methods

This retrospective study received our institution’s review board approval (protocol 23-63: impact of extraventricular drain care bundle on infection rate associated with extraventricular drain) and was conducted from June 2022 to June 2023 at a single center. Patient consent was waived given the retrospective nature of the study. Electronic medical records were used to identify patients who underwent bedside EVD insertion in the intensive care unit by using the Current Procedural Terminology (CPT) code 61107 [7]. Patients who underwent EVD insertion in the emergency department or operating room were excluded from the study since the EVD care bundle was not used. First, comparisons of patient characteristics and outcomes were made between patients who did not have CSF infection and patients who had CSF infection in 2022. Then, comparisons of patient characteristics and outcomes were made before and after the implementation of the EVD care bundle. The primary outcome was to compare the infection rate before and after the implementation of the EVD care bundle. To achieve this, the CSF profiles and antibiotic usage were evaluated. Infection was defined as positive CSF cultures that required treatment. The secondary outcomes included EVD-related procedures (EVD insertion, EVD replacements, CSF sampling, and intrathecal medication installation), length of EVD dwelling, and systemic infection. Systemic infection was defined as positive blood, respiratory, or urine cultures that required treatment. Data were analyzed using the GraphPad Software (GraphPad Software, Boston, MA) [8]. T-test, Fisher’s exact test, and two-way analysis of variance (ANOVA) test were used. A p-value <0.05 was considered statistically significant. 

## Results

Without an EVD care bundle 

**Figure 1:**
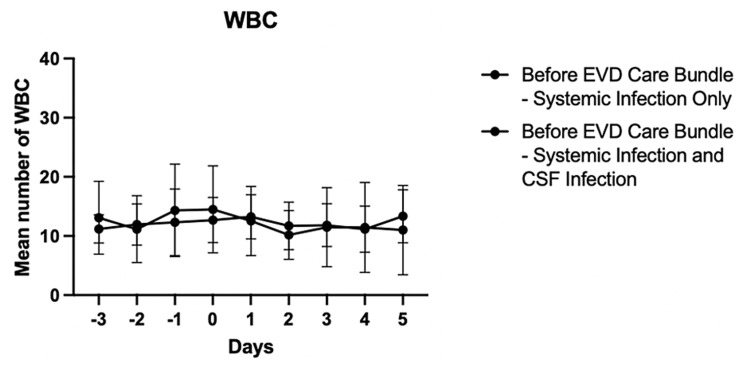
Comparison of WBC count between patients with systemic infection only and patients with systemic infection + CSF infection from June 2022 to December 2022 X-axis: Zero is the date of culture collection for systemic infection, not CSF collection. Negative numbers are days prior to the date of culture collection and positive numbers are days after the date of culture collection. WBC: white blood cell; EVD: extraventricular drain; CSF: cerebrospinal fluid.

**Figure 2:**
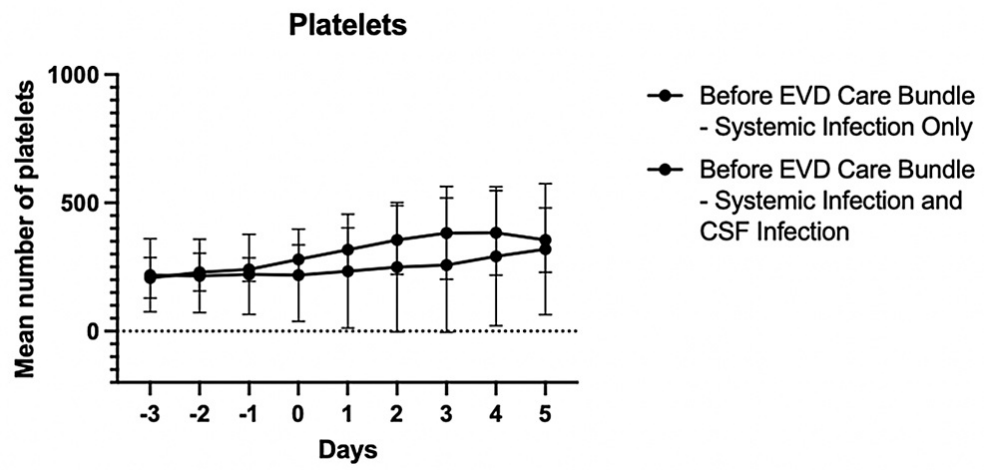
Comparison of platelet number between patients with systemic infection only and patients with systemic infection + CSF infection from June 2022 to December 2022 X-axis: Zero is the date of culture collection for systemic infection, not CSF collection. Negative numbers are days prior to the date of culture collection and positive numbers are days after the date of culture collection. EVD: extraventricular drain; CSF: cerebrospinal fluid.

Prior to the implementation of the EVD care bundle in January 2023, there were a total of 33 patients, 273 EVD-related procedures, and 581 catheter days. The CSF infection rate was 21.2% (7/33 patients). The mean age of these seven patients was younger than that of the 26/33 patients who did not have CSF infection (34.9 years vs. 52.1 years, respectively, p=0.0418) (Table 1). The total mean number of EVD-related procedures including EVD placements, EVD replacements, CSF sampling, and intrathecal medication installation were not statistically different (CSF infection: 9.4 vs. no CSF infection: 8.0, p=0.7364). There was a higher mean number of EVD placements and replacements in the seven patients with CSF infection (1.4 vs. 3.4, p=0.0028). The mean interval time between initial EVD placement and positive CSF culture was 15.9 days in the seven patients with CSF infection, which was not statistically different from 12.2 days of negative CSF culture results in the 26 patients without CSF infection. The total length of EVD dwelling was significantly higher in the seven patients with CSF infection compared to no infection (37.6 days vs. 12.2 days, p<0.0001). The CSF cultures grew *Acinetobacter ursingii, Enterobacter colace complex, Stentotrophomonas matophilia, Enterococcus faecium, Staphylococcus epidermidis, Methicillin-resistant Staphylococcus aureus,* and *Klebsiella pneumoniae extended-spectrum beta-lactamases*. All seven patients with CSF infection were treated with intrathecal and intravenous antibiotics. There were a total of 11/33 patients without systemic infection and all 11 patients did not have CSF infection (Table 2). On the other hand, of the 22/33 patients with systemic infection, seven of them had CSF infection. The most common source of systemic infection was respiratory (18/22, 82%). There was no difference in the mean WBC count and the mean platelet number between patients with systemic infection only and patients with systemic infection + CSF infection (WBC: p=0.9998 and platelets: p=0.3832) (Figures 1, 2). 

**Table 1:** Patient characteristics and interventions before EVD care bundle The data has been represented as N, %, and mean ± SD. A p-value <0.05 is considered statistically significant. CSF: cerebrospinal fluid; N: number of patients; SD: standard deviation; EVD: extraventricular drain.

Parameter		No CSF infection (N = 26)	CSF Infection (N = 7)	p-Value
Age (years), mean ± SD		52.12 ± 20.27	34.86 ± 13.02	0.0418*
Sex, N (%)	Female	8 (30.77%)	1 (14.29%)	0.6418
	Male	18 (69.23%)	6 (85.71%)	
EVD replacements, mean ± SD		1.39 ± 0.75	3.43 ± 3.00	0.0028*
CSF sampling, mean ± SD		3.23 ± 3.17	5.14 ± 4.63	0.2088
Intrathecal medication instillation, mean ± SD		3.35 ± 9.25	0.86 ± 1.57	0.4883
Total number of EVD replacement, CSF sampling, and intrathecal medication instillation, mean ± SD		7.96 ± 10.63	9.43 ± 7.79	0.7364
Interval between date of EVD placement and positive CSF culture (days), mean ± SD		12.23 ± 9.75	15.29 ± 13.92	0.5071
Total length of EVD duration (days), mean ± SD		12.23 ± 9.75	37.57 ± 22.14	<0.0001*

**Table 2:** Relationship between CSF Infection and systemic Infection from June 2022 to December 2022 The data has been represented as N and %. No statistical analysis was performed. CSF: cerebrospinal fluid; N: number of patients.

Parameter		No CSF Infection (N = 26)	CSF Infection (N = 7)
No systemic infection, N (%)		11 (42.31%)	0 (0.00%)
Systemic infection, N (%)		15 (57.69%)	7 (100.00%)
Systemic infection: positive culture type, N (%)	Blood culture	1 (6.67%)	2 (28.57%)
	Respiratory culture	13 (86.67%)	5 (71.43%)
	Urine culture	1 (6.67%)	0 (0.00%)

With an EVD care bundle

After the implementation of the EVD care bundle, there were a total of 25 patients, 131 EVD-related procedures, and 267 catheter days. Therefore, from June 2022 to June 2023, a total of 58 patients, 404 EVD-related procedures, and 848 catheter-days were evaluated. The mean age was 48.5 years in 2022 and 48.2 years in 2023 (p=0.9663) (Table 3). Twenty-four of 33 patients (72.7%) and 14/25 patients (56.0%) were male, respectively (p=0.2653). The total mean number of EVD-related procedures including EVD placements, EVD replacements, CSF sampling, and intrathecal medication installation were not statistically different (2022: 8.3 vs. 2023: 5.2, respectively, p=0.1892). However, the mean number of EVD placements and replacements was statistically significantly less in 2023 (1.8 vs. 1.0, respectively, p=0.0257) compared to 2022 when there were infections. The mean total EVD dwelling was 17.6 days in 2022 and 10.7 days in 2023 (p=0.0639). The CSF infection rate was 21.2% (7/33 patients) before the implementation of the EVD care bundle and zero (0/25 patients) afterward. There were a total of 13/25 patients in 2023 with systemic infection and 12/25 patients without systemic infection and none of them had CSF infection (Table 4). The most common source of 2023 systemic infection was respiratory (11/13, 85%). 

**Table 3:** Patient characteristics and interventions before and after EVD care bundle The data has been represented as N, %, and mean ± SD. A p-value <0.05 is considered statistically significant. N: number of patients; SD: standard deviation; EVD: extraventricular drain; CSF: cerebrospinal fluid.

Parameter		Before (N=33)	After (N=25)	p-Value
Age (years), mean ± SD		48.45 ± 20.11	48.24 ± 17.59	0.9663
Sex, N (%)	Female	9 (27.27%)	11 (44.00%)	0.2653
	Male	24 (72.73%)	14 (56.00%)	
EVD replacements, mean ± SD		1.82 ± 1.69	1.04 ± 0.20	0.0257*
CSF sampling, mean ± SD		3.75 ± 3.53	2.84 ± 4.23	0.3798
Intrathecal medication instillation, mean ± SD		2.82 ± 8.27	1.36 ± 3.11	0.4063
Total number of EVD replacement, CSF sampling, and intrathecal medication instillation, mean ± SD		8.27 ± 10.00	5.24 ± 6.28	0.1892
Total length of EVD duration (days), mean ± SD		17.61 ± 16.64	10.68 ± 8.75	0.0639

**Table 4:** Relationship between CSF infection and systemic infection: before vs. after EVD care bundle The data has been represented as N and %. No statistical analysis was performed. N: number of patients; CSF: cerebrospinal fluid.

Parameter			Before (N=33)	After (N=25)
No CSF infection, N (%)			26 (78.79%)	25 (100.00%)
No CSF infection without systemic infection			11 (42.31%)	12 (48.00%)
No CSF infection with systemic infection, N (%)			15 (57.69%)	13 (52.00%)
No CSF infection with systemic infection: positive culture type, N (%)		Blood culture	1 (6.67%)	0 (0.00%)
		Respiratory culture	13 (86.67%)	11 (84.62%)
		Urine culture	1 (6.67%)	2 (15.38%)
CSF infection, N (%)			7 (21.21%)	0 (0.00%)
CSF infection without systemic infection, N (%)			0 (0.00%)	0 (0.00%)
CSF infection with systemic infection, N (%)			7 (100.00%)	0 (0.00%)
CSF infection with systemic infection: positive culture type, N (%)	Blood culture		2 (28.57%)	0 (0.00%)
	Respiratory culture		5 (71.43%)	0 (0.00%)
	Urine culture		0 (0.00%)	0 (0.00%)

## Discussion

**Figure 3:**
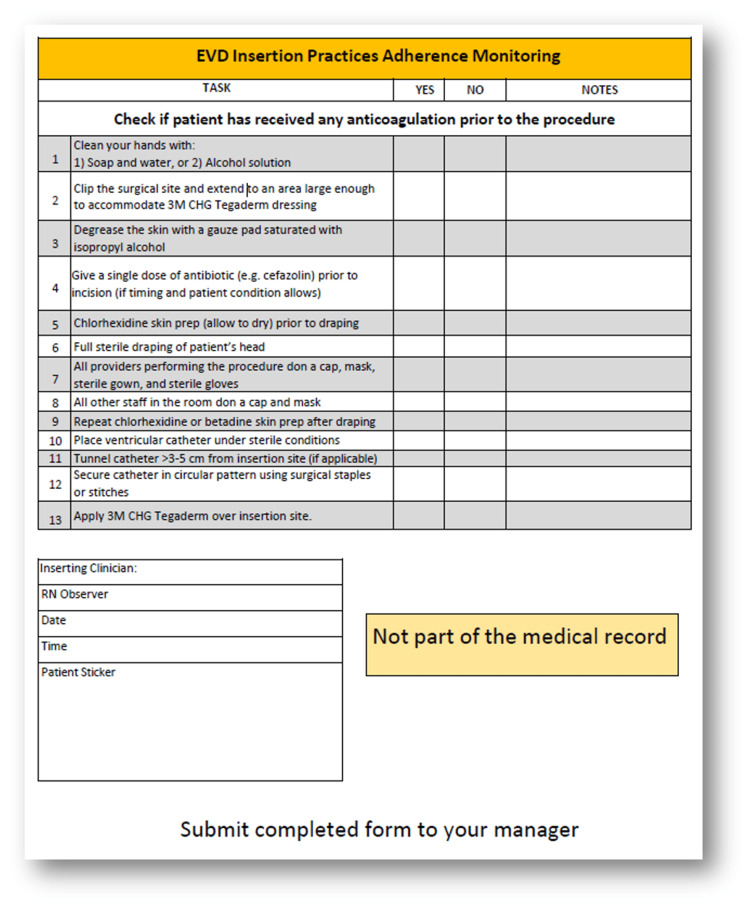
EVD care bundle checklist: EVD insertion practices adherence monitoring EVD: extraventricular drain.

**Figure 4:**
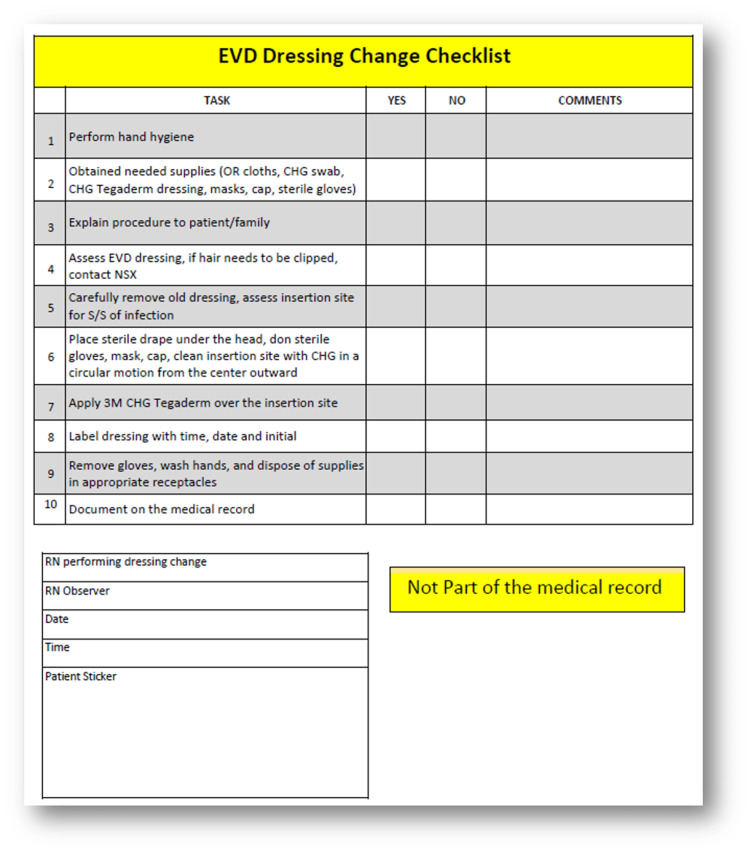
: EVD care bundle checklist: EVD dressing change checklist EVD: extraventricular drain.

**Figure 5:**
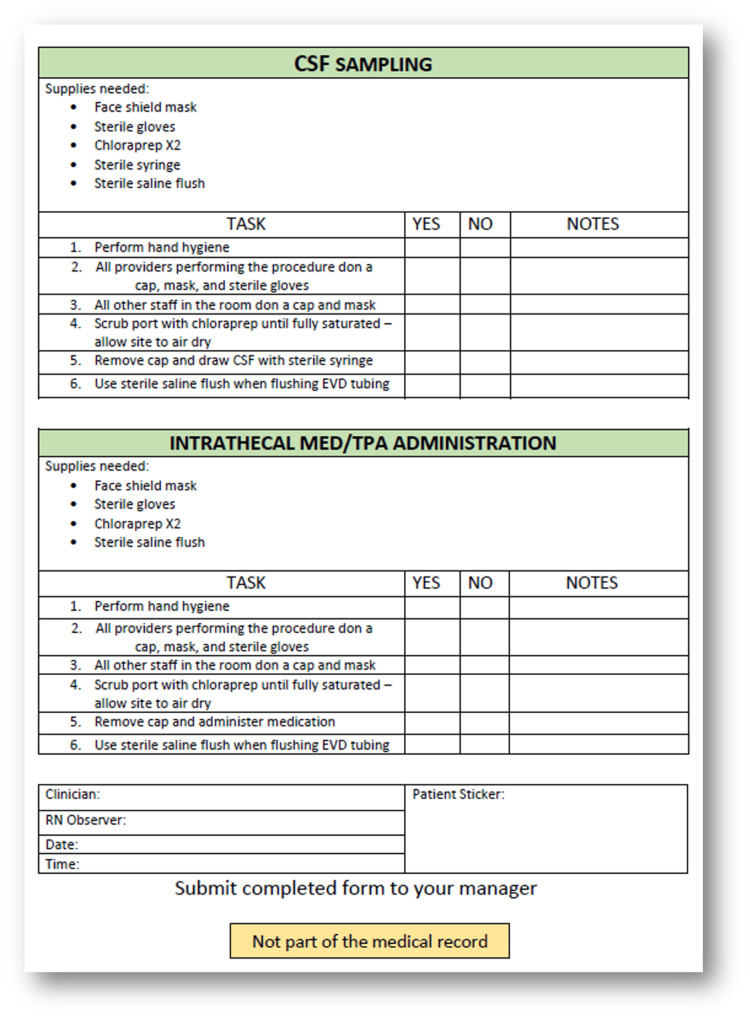
EVD care bundle checklists: CSF sampling and intrathecal med/tPA administration EVD: extraventricular drain; CSF: cerebrospinal fluid; med: medication; tPA: tissue plasminogen activator.

EVD placement is a commonly performed bedside procedure and infection rates associated with EVD have been reported to be 3.9%-18.5% [9,10]. Our overall infection rate prior to the implementation of the EVD care bundle was 21.2%, which is higher than reported. With the implementation of the EVD care bundle, the infection rate was successfully reduced to 0.0%. Strict adherence to three of the four EVD care bundle checklists (EVD insertion, CSF sampling, and intrathecal medication instillation) was observed because they require the presence of both physician and nurse (Figures 3-5 in Appendices). However, the EVD dressing change checklist compliance was inconsistent as not all nursing staff reliably documented the procedure when performed by a colleague; this gap in documentation hinders the effective evaluation of adherence to the checklist.

The concept of an EVD care bundle to reduce infection rates is not novel. The main objective of such a bundle is to standardize practices, utilize checklists, and perform real-time monitoring including insertion technique, prophylactic antibiotic use, antibiotic-impregnated catheter use, limitations on CSF sampling and catheter access, and sterile dressing [11]. Talibi et al. and Kubilay et al. found a decrease in infection rates after the implementation of the EVD care bundle [5,6]. Our data align with their findings. EVD care bundle also helps create a culture of safety [6]. 

Previously, Camacho et al. found that the duration of EVD dwelling was the only independent risk factor associated with the infection rate [12]. While the total duration of EVD dwelling was longer in those patients with CSF infection, it was found to be due to EVD being left in place for intrathecal antibiotics installation. There was no difference in duration from EVD insertion to positive CSF culture between patients with CSF infection and those without CSF infection. This demonstrated that the duration of EVD dwelling prior to positive CSF culture was not a contributing factor to CSF infection. 

Our data shows that the total number of EVD-related procedures which includes EVD placements, CSF sampling, and intrathecal medication installation was not higher in patients who had CSF infection. However, there were also more EVD replacements in patients who had CSF infection since most positive infections resulted in an EVD change. Other reasons for EVD change include blockage and malposition. This suggests that the types of EVD-related procedures are not equivalent in terms of infection risk. During 2022, EVD replacement without prior infection, not CSF sampling or intrathecal medication installation, was associated with a higher risk of CSF infection. The higher risk of infection can be attributed to the increased technical complexity involved in the EVD replacement procedure. Data analysis reveals a correlation between the frequency of EVD replacements and the incidence of CSF infections. This finding underscores the importance of implementing a standardized procedure, checklist, and real-time monitoring for EVD replacements. Such a bundle is instrumental in maintaining rigorous aseptic techniques during the EVD replacement process, potentially reducing the risk of infection. Implementing the EVD care bundle enhances teamwork across multiple disciplines, fosters a sense of joint responsibility through the employment of standardized checklists, and cultivates a safety-centric environment.

Our analysis identified a link between systemic and CSF infections. Notably, before the EVD care bundle's implementation, 68.2% of patients with systemic infections were free of CSF infections. This percentage rose to 100% after implementation, indicating that systemic infections likely weaken the immune system, increasing the susceptibility to CSF infections. However, the introduction of the EVD care bundle significantly mitigated this risk, suggesting its effectiveness in preventing CSF infections in patients already battling systemic infections.

Often blood leukocytosis and thrombocytopenia are present in severe systemic infection [13,14]. Overall, on average, blood leukocytosis was observed in our patients with systemic infection and CSF infection. 

There are some limitations to our study. This is a single institution’s experience and also a retrospective study of one year. This study evaluates the impact of an EVD care bundle in EVD-related procedures. There are no other care bundles for other procedures such as lumbar drain placement and intracranial oxygen or temperature monitoring devices. Future studies should consider developing care bundles for other procedures and investigate the impact of these care bundles on other procedures over a longer period of time in prospective studies. Furthermore, implementation of care bundles should not be limited to neurosurgical procedures and we encourage other specialties and institutions to consider using care bundles for non-neurosurgical procedures as well. 

## Conclusions

EVD-related procedures are associated with complications including infection. EVD replacements and systemic infection, not EVD initial insertion, CSF sampling, or intrathecal medication installation, were found to be associated with CSF infection. The implementation of the EVD care bundle reduced the infection rate from 21.2% to 0.0% over one year. We therefore encourage the use of EVD care bundle for procedure standardization, checklists, and real-time procedure adherence monitoring.
